# 通过机器学习探究外周血相关指标对非小细胞肺癌*EGFR*突变及预后的预测价值研究

**DOI:** 10.3779/j.issn.1009-3419.2025.102.05

**Published:** 2025-03-21

**Authors:** Shulei FU, Shaodi WEN, Jiaqiang ZHANG, Xiaoyue DU, Ru LI, Bo SHEN

**Affiliations:** ^1^210009 南京，南京医科大学附属肿瘤医院/江苏省肿瘤医院/江苏省肿瘤防治研究所; ^1^The Affiliated Cancer Hospital of Nanjing Medical University, Jiangsu Cancer Hospital, Jiangsu Institute of Cancer Research,Nanjing 210009, China; ^2^010000 呼和浩特，内蒙古大学; ^2^Inner Mongolia University, Hohhot 010000, China

**Keywords:** 肺肿瘤, 表皮生长因子受体, 机器学习, 预测模型, Lung neplasms, Epidermal growth factor receptor, Machine learning, Predictive model

## Abstract

**背景与目的:**

表皮生长因子受体（epidermal growth factor receptor, EGFR）敏感突变是非小细胞肺癌（non-small cell lung cancer, NSCLC）靶向治疗的有效靶点之一。然而，由于部分原发组织难以获取及部分经济欠发达地区经济因素，部分患者无法进行传统基因检测。本研究旨在利用非侵入性的外周血指标，建立机器学习（machine learning, ML）模型，探索NSCLC中与EGFR突变状态密切相关的生物标志物，并评估其在预后中的潜在价值。

**方法:**

回顾性地收集2016年11月至2023年5月就诊于江苏省肿瘤医院的2642例肺癌患者的临床指标，将有完整随访数据的175例NSCLC患者纳入研究。根据外周血指标构建ML模型，按照8:2的比例分为训练集和测试集。采用无监督学习算法对血液特征进行聚类，使用互信息法进行特征筛选，并设计基于Shapley值的集成学习算法，计算每个特征对于模型预测结果的贡献程度。使用受试者工作特征（receiver operating characteristic, ROC）曲线对模型预测能力进行评估。

**结果:**

通过基于Shapley值的可解释ML模型的特征提取和对预测结果的贡献度分析，筛选出前10个贡献度最高的指标，分别为：病理类型、磷、嗜酸性粒细胞、单核细胞计数、活化部分凝血活酶时间、钾、总胆红素、钠、嗜酸性粒细胞百分比及总胆固醇。本研究模型的曲线下面积（area under the curve, AUC）为0.80。此外，低血钠及病理类型为鳞癌组的患者预后较差（P<0.05）。

**结论:**

本研究构建的可解释的模型为NSCLC患者EGFR突变状态的预测提供了新方法，这对无法进行基因检测的患者的诊疗提供了较为科学的依据。

## Abstract (en)

**Background and objective:**

Epidermal growth factor receptor (EGFR) sensitive mutation is one of the effective targets of targeted therapy for non-small cell lung cancer (NSCLC). However, due to the difficulty of obtaining some primary tissues and the economic factors in some underdeveloped areas, some patients cannot undergo traditional genetic testing. The aim of this study is to establish a machine learning (ML) model using non-invasive peripheral blood markers to explore the biomarkers closely related to EGFR mutation status in NSCLC and evaluate their potential prognostic value.

**Methods:**

2642 lung cancer patients who visited Jiangsu Cancer Hospital from November 2016 to May 2023 were retrospectively enrolled and finally 175 NSCLC patients with complete follow-up data were included in the study. The ML model was constructed based on peripheral blood indicators and divided into training set and test set according to the ratio of 8:2. Unsupervised learning algorithms were used for clustering blood features and mutual information method for feature selection, and an ensemble learning algorithm based on Shapley value was designed to calculate the contribution of each feature to the model prediction result. The receiver operating characteristic (ROC) curve was used to evaluate the predictive ability of the model.

**Results:**

Through the feature extraction and contribution analysis of the predictive results of the interpretable ML model based on the Shapley value, the top ten indicators with the highest contribution were: pathological type, phosphorus, eosinophils, monocyte count, activated partial thromboplastin time, potassium, total bilirubin, sodium, eosinophil percentage, and total cholesterol. The area under the curve (AUC) of the model was 0.80. In addition, patients with hyponatremia and squamous cell carcinoma group had a poor prognosis (P<0.05).

**Conclusion:**

The interpretable model constructed in this study provides a new approach for the prediction of EGFR mutation status in NSCLC patients, which provides a scientific basis for the diagnosis and treatment of patients who cannot undergo genetic testing.

在最新的全球癌症统计中，肺癌的患病率和死亡率仍位居首位^[1]^。非小细胞肺癌（non-small cell lung cancer, NSCLC）是最常见的组织学亚型，约占所有患者的85%。肺癌的治疗根据肿瘤分期、患者身体状况和基因突变等因素而定。针对表皮生长因子受体（epidermal growth factor receptor, EGFR）和间变性淋巴瘤激酶（anaplastic lymphoma kinase, ALK）等基因突变的靶向药物已在特定患者群体中取得显著疗效^[2]^。近20%的白种人和高达50%的亚洲人肺腺癌患者携带EGFR基因突变^[3]^。EGFR基因突变会导致EGFR下游信号转导的配体非依赖性激活，从而促进细胞增殖、存活和迁移^[4]^。几乎90%的EGFR突变由19外显子缺失和21外显子点突变组成^[5]^。据统计，在多个欧洲国家（2011至2016年），接受分子检测的晚期非鳞状NSCLC患者的比例在65%-85%之间^[6]^。这种状况凸显了进一步提高EGFR基因检出率的需要，以确保所有晚期非鳞状NSCLC患者能及时获得精准医疗。EGFR突变在肺癌中具有重要的临床意义，可用于预测患者的预后、指导靶向治疗选择，并帮助监测治疗反应和耐药性的发展，这对指导临床诊疗具有重要意义。

复杂的肿瘤微环境可以通过多种方式影响循环中生物标志物的变化，外周血中生物标志物对环境变化极为敏感，可以快速对肿瘤状态、治疗效果和预后等做出反应，并为肿瘤治疗策略的选择和监测提供依据。研究^[7]^表明，肺腺癌患者EGFR基因突变状态和外周血中性粒细胞数、中性粒细胞与淋巴细胞比值存在相关性，可预测肺腺癌患者的EGFR基因突变状态。此外，马娟团队^[8]^的研究进一步探讨了血脂谱与EGFR T790M突变之间的潜在联系，发现高载脂蛋白A1水平与EGFR T790M突变呈正相关，而治疗前外周血中高密度脂蛋白胆固醇和载脂蛋白A1的高水平则预示着EGFR-酪氨酸激酶抑制剂（EGFR-tyrosine kinase inhibitors, EGFR-TKIs）治疗后的患者可能获得更长的无进展生存期（progression-free survival, PFS）。

目前临床常用的基因突变检测方法为病理穿刺组织和外周血基因检测，但两者存在各自的局限性。前者可能会受到组织样本不足、活检取样困难以及肿瘤转移等限制。后者则因血浆中肿瘤循环DNA（tumor circulating DNA, ctDNA）数量有限、半衰期短等导致假阴性结果高，该检测方法较穿刺检出率低且对技术要求高^[9]^，并且较长的检测周转时间往往导致无法及时进行术前指导和治疗策略的制定。鉴于此，开发一种经济高效、操作简便的EGFR突变预测方法，对于资源匮乏地区的医疗机构及对侵入性检查耐受性差的患者而言，具有重要的临床价值。本研究通过收集NSCLC患者的完整外周血数据，构建机器学习（machine learning, ML）模型进行深入分析，寻找与EGFR突变状态密切相关的生物标志物，对无法进行基因检测的患者的诊疗提供科学依据，并评估其在预后中的潜在价值。

## 1 资料与方法

### 1.1 研究对象

回顾性收集2016年11月至2023年5月就诊于江苏省肿瘤医院的2642例肺癌患者的临床指标，将有完整随访数据的175例NSCLC患者纳入研究。纳入标准：（1）诊断为NSCLC；（2）年龄>18岁；（3）发生EGFR突变的患者，突变类型为19外显子缺失或21外显子点突变，且不存在EGFR合并突变（EGFR双位点或其他有临床诊疗意义的基因突变）；（4）病理类型为腺癌或鳞癌；（5）体内至少有1个可以进行评价的肿瘤病灶；（6）有完整的血液学检测指标。排除标准：（1）临床资料不完整；（2）肺癌复发或者其他部位原发肿瘤转移到肺部；（3）伴发具有十分明显的其他重要脏器功能缺陷；（4）特殊人群，如孕妇、哺乳期妇女等。

### 1.2 一般资料

通过电子病历系统收集患者的临床指标，包括病史资料（年龄、性别、吸烟史、放疗史、手术史等）、血液学资料（血常规、生化、肿瘤标志物、凝血功能、甲状腺功能检测等）、病理类型、初次确诊时间、肿瘤分期、驱动基因突变状态、美国东部肿瘤协作组体能状态（Eastern Cooperative Oncology Group performance status, ECOG PS）评分等。肿瘤分期依据美国癌症联合委员会（American Joint Committee on Cancer, AJCC）第八版定义的肺癌分期。本研究由江苏省肿瘤医院机构评审委员会批准。

### 1.3 ML方法

#### 1.3.1 特征工程及模型建立

本研究使用Python构建ML模型。为探究外周血指标与NSCLC患者EGFR突变之间的关联，本研究对NSCLC患者的常规血液数据进行了统计分析，以构建血液成分和基因突变之间的关联网络。首先进行数据的预处理，针对样本不平衡问题，采用过采样算法，尽可能实现平衡的样本分布。此外，考虑到多达100个血液学变量，采用无监督聚类学习方法深度探索数据内在结构并发现隐藏信息。本研究采用K-Means算法，对外周血样本的特征进行聚类，通过迭代更新簇内数据点与簇中心的距离进行聚类，从而对这些变量进行分类，并识别与基因突变有强相关性的组。受信息熵的启发，利用互信息法（互信息回归或信息增益）进行特征筛选，引入互信息值来衡量两个随机变量之间的相关性。互信息值越大，表示两随机变量之间的相关性越高。基于互信息值对特征进行排序，筛选出一定数量性能最佳的特征。具体来说，将血液学的100多个变量定义为X，基因突变定义为Y。由于每个变量X是离散的，根据每个Y的预测频率来计算离散变量X的边际概率。对于X和Y的联合分布概率，假设它们相对独立，可以直接计算边际概率的乘积。P（Y=y）的边际概率是通过对血液学变量的所有可能组合求和来计算的，有了上述条件，就可以列出等式计算^[10,11]^。将ML模型设置为f（x, y），其中模型输出结果为S（x, y），用于单流预测计算。然后使用XGBoost算法训练模型，在训练过程中，对模型的超参数进行调整，包括树的最大深度、学习率和树的数量等，以确保模型既不会过拟合也不会欠拟合。实验过程中数据集以8:2的比例分为训练集和测试集。

#### 1.3.2 可解释性分析与模型性能评估

ML模型因其内部决策过程复杂且难以解释，往往被视为“黑箱”，这种“黑箱”现象限制了模型在实际应用中的信任度和可接受性，为了最大限度地提高透明度，引入改进后的Shapley值近似算法来揭示模型的内部机制。其在ML和科学数据中被广泛应用于解释模型的预测结果，特别是用于量化每个特征对模型预测的贡献。为了计算血液特征的贡献度，确保特征贡献的公平分配，同时考虑到原始算法的时间复杂度较高，引入蒙特卡洛近似来估计Shapley值，即采用随机抽样的方式计算每个特征的贡献度^[12]^。具体而言，记样本为x_i_，预测值为y_i_，基线预测值为，x_i_^j^为样本i的第j个特征，可认为样本的预测值y_i_由基线预测值与每个特征的贡献x_i_^j^组成。其中为单特征均值。

而单特征的贡献由近似下的Shapley值计算，如下：

其中m是蒙特卡洛采样次数，S_k_是k次随机选择的特征子集，v（-）是价值。重复采样多次即可计算出特征的平均贡献度。由此筛选出最大的前10个特征，分别对这10个特征进行单特征预测，并且又随机从100多个特征中抽取数次10个特征进行预测，与贡献度筛选出的10个特征预测进行对比，结果显示，单特征预测和随机抽样数次所获得的特征均没有筛选出的10个特征预测效果好。本研究使用受试者工作特征（receiver operating characteristic, ROC）曲线评估模型的诊断价值，展示模型在不同决策阈值下的表现。

### 1.4 统计学方法

采用R 4.2.3软件进行统计分析，使用CBCgrps v2.8软件包判断变量的类型和分布，并进行统计描述和双变量分析。计量资料用Shapior-Wilk test检验正态性，符合正态分布的连续变量用均数±标准差表示，如满足方差齐性则组间比较采用独立样本t检验，反之采用秩和检验；不符合正态分布的连续变量以中位数和四分位间距表示，组间比较采用Mann-Whitney U检验；计数资料用频数（百分比）表示，组间比较采用卡方检验或确切概率法。应用X-Tile软件计算与EGFR突变相关性排名前10标志物的最佳截断值；若任意一组占比小于30%，则采用中位数分组。结局指标为在对患者进行末次随访时，患者是否存活。通过Kaplan-Meier法计算中位随访时间。依据与EGFR突变相关性最强的临床指标绘制生存曲线。考虑到临床分期对患者生存及预后的影响，进行生存分析时将患者进行分层生存分析，因早期NSCLC患者占比较小，使用对数秩检验（Log-rank检验）通过危险比（hazard ratio, HR）进行比较。以P<0.05为差异具有统计学意义（双侧检验）。

## 2 结果

### 2.1 研究对象的临床特征

本研究共175例NSCLC患者被纳入研究。其中，中位年龄64.00（57.50-69.50）岁，男性111例（63.43%），女性64例（36.57%）；EGFR突变型83例（47.43%），野生型92例（52.57%）；鳞癌45例（25.71%），腺癌130例（74.29%）。腺癌患者中83例（63.85%）伴有EGFR突变，鳞癌患者中无EGFR突变。性别、病理类型、是否手术、是否发生远处转移、ECOG PS、是否吸烟等指标在突变组与野生组具有统计学差异（P<0.05）（表1）。并对纳入的患者进行随访，末次随访时间为2024年4月3日。收集到结局的患者为146例（83.43%），但因17例患者未收集到确切的死亡时间被排除在外，最终纳入129例（73.71%）有完整随访时间的患者。其中40例患者死亡，89例存活，最短随访时间为11个月，最长随访时间为88个月。中位随访时间35.0个月、中位总生存期56个月（2-88个月）。

**表1:** 根据EGFR突变状态分组的NSCLC患者的临床特征

Variables	Total (n=175)	Non-mutated (n=92)	Mutated (n=83)	P
Age (yr)	64.00 (57.50-69.50)	64.50 (57.75-70.25)	63.00 (57.50-68.00)	0.13
Gender				<0.01
Male	111 (63.43%)	73 (79.35%)	38 (45.78%)	
Female	64 (36.57%)	19 (20.65%)	45 (54.22%)	
Pathology				<0.01
SCC	45 (25.71%)	45 (48.91%)	0 (0.00%)	
Adeno	130 (74.29%)	47 (51.09%)	83 (100.00%)	
TNM stage				0.27
I-II	35 (20.00%)	15 (16.30%)	20 (24.10%)	
III-IV	140 (80.00%)	77 (83.70%)	63 (75.90%)	
Distant metastasis				0.03
Yes	91 (52.00%)	40 (43.48%)	51 (61.45%)	
No	84 (48.00%)	52 (56.52%)	32 (38.55%)	
Operation				0.02
Yes	33 (18.90%)	11 (11.96%)	22 (26.51%)	
No	142 (81.10%)	81 (88.04%)	61 (73.49%)	
Radiotherapy				0.67
Yes	5 (2.90%)	2 (2.17%)	3 (3.61%)	
No	170 (97.10%)	90 (97.82%)	80 (96.39%)	
ECOG PS				0.04
0	76 (43.43%)	34 (36.96%)	42 (50.60%)	
1	99 (56.57%)	58 (63.04%)	41 (49.40%)	
Smoking				<0.01
Yes	22 (12.57%)	20 (21.74%)	2 (2.41%)	
No	153 (87.43%)	72 (78.26%)	81 (97.59%)	

NSCLC: non-small cell lung cancer; EGFR: epidermal growth factor receptor; SCC: squamous carcinoma; Adeno: adenocarcinoma; TNM: tumor-node-metastasis; ECOG PS: Eastern Cooperative Oncology Group performance status.

### 2.2 分析结果

#### 2.2.1 基于ML临床预测模型输出与EGFR突变相关的生物标志物

通过ML模型的分析，识别出与EGFR突变相关性贡献最高的前10个临床指标，分别为：病理类型、磷、嗜酸性粒细胞、单核细胞计数、活化部分凝血活酶时间（activated partial thromboplastin time, APTT）、钾、总胆红素、钠、嗜酸性粒细胞百分比和总胆固醇。整体模型的曲线下面积（area under the curve, AUC）为0.80，剔除病理类型后AUC为0.83，显示出较好的预测性能。数据集按8:2比例分为训练集和测试集，具体AUC见表2。使用ROC曲线评估10个指标的预测性能（图1）。本研究设计考虑多个指标之间的相互补充和综合效应，因而整体的预测效能应远优于各单项指标。通过比较整体与各指标的ROC曲线及AUC值，结果与之吻合。

**表2:** 通过机器学习确定的贡献最高的前10个特征及其AUC值

Characteristic	Train group	Test group
All	1.00	0.80
Pathology	0.75	0.75
Phosphorus	0.91	0.50
Eosinophils	0.80	0.59
Monocytes	0.89	0.62
Activated partial thromboplastin time	0.89	0.63
Potassium	0.92	0.64
Total bilirubin	0.92	0.56
Sodium	0.88	0.55
Eosinophil percentage	0.89	0.62
Total cholesterol	0.95	0.52

AUC: area under the curve.

**图1:**
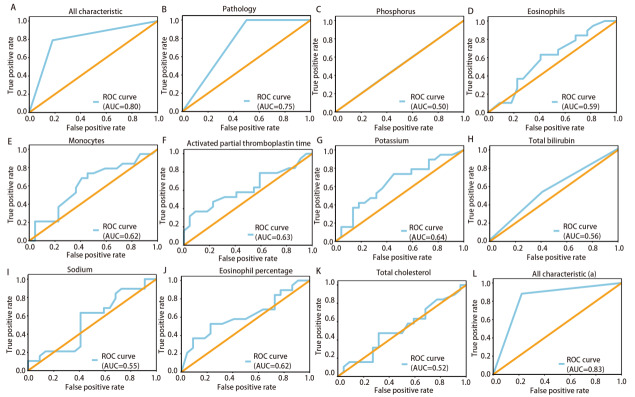
贡献最高的前10个特征的ROC曲线。A：所有特征；B：病理；C：磷；D：嗜酸性粒细胞；E：单核细胞；F：活化部分凝血活酶时间；G：钾；H：总胆红素；I：钠；J：嗜酸性粒细胞百分比；K：总胆固醇；L：所有特征（a）：排除病理类型后的ROC曲线。

#### 2.2.2 基于相关的生物标志物对NSCLC患者的生存分析

利用ML模型识别出与EGFR突变相关性贡献最高的10个临床指标，为了进一步评估这些指标对NSCLC患者预后的影响，进行生存分析，以直观展示不同指标水平对患者生存率的影响。整体分析的结果显示，在病理类型方面，与腺癌患者相比，鳞癌患者的预后较差（P=0.01）；低血钠组（血钠<142.7 mmol/L）及未发生EGFR突变的NSCLC患者与较差的预后有关（P<0.05，图2）。通过依据肿瘤分期进一步将NSCLC患者进行分层生存分析。晚期NSCLC患者（III-IV期）共102例（79.07%），通过生存分析后与整体的结果相比较，仅EGFR的突变状态与预后有关，即EGFR未突变组预后较差（P=0.02，图3）。早期NSCLC患者（I-II期）则采用HR值及95%CI来表示（表3），其中，病理类型、血钠及总胆固醇变量中因死亡病例均位于同一组，因而未得出HR值。通过对早期NSCLC患者生存分析，未发现存在统计学意义上与预后有关的组别。

**图2:**
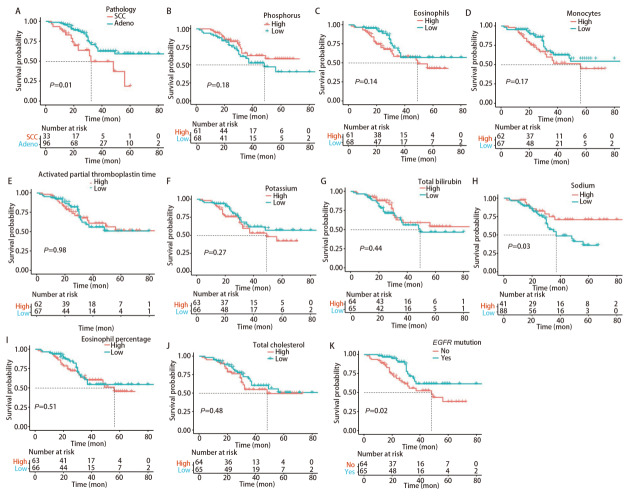
贡献最高的前10个特征及EGFR突变状态的生存曲线图。A：病理；B：磷；C：嗜酸性粒细胞；D：单核细胞；E：活化部分凝血活酶时间；F：钾；G：总胆红素；H：钠；I：嗜酸性粒细胞百分比；J：总胆固醇；K：EGFR突变状态。

**图3:**
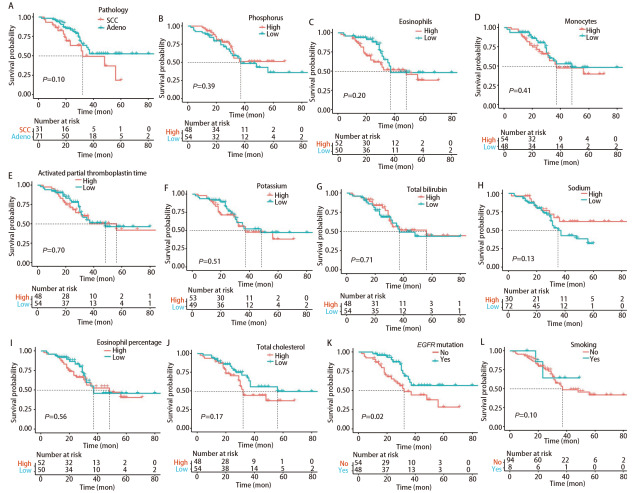
晚期肺癌患者中贡献最高的前10个特征及EGFR突变状态、吸烟史的生存曲线图。A：病理；B：磷；C：嗜酸性粒细胞；D：单核细胞；E：活化部分凝血活酶时间；F：钾；G：总胆红素；H：钠；I： 嗜酸性粒细胞百分比；J：总胆固醇；K：EGFR突变状态；L：吸烟情况。

**表3:** 早期肺癌中及EGFR突变状态贡献最高的前10个特征的风险比

Characteristic	HR (95%CI)	P
Pathology		
SCC vs Adeno	-	>0.99
Phosphorus (mmol/L)		
≥1.18 vs <1.18	3.99 (0.36-44.01)	0.20
Eosinophils (/L)		
≥0.12×10^9^ vs <0.12×10^9^	1.26 (0.12-12.84)	0.85
Monocytes (/L)		
≥0.49×10^9^ vs <0.49×10^9^	0.70 (0.05-9.27)	0.77
Activated partial thromboplastin time (s)		
≥26.50 vs <26.50	1.08 (0.09-12.33)	0.95
Potassium (mmol/L)		
≥4.20 vs <4.20	0.43 (0.05-4.23)	0.48
Total bilirubin (μmol/L)		
≥8.90 vs <8.90	2.77 (0.28-27.41)	0.38
Sodium (mmol/L)		
≥142.70 vs <142.70	-	0.09
Eosinophil percentage		
≥1.80% vs <1.80%	1.50 (0.15-14.78)	0.74
Total cholesterol (mmol/L)		
≥4.44 vs <4.44	-	0.09
EGFR mutation status		
Non-mutated vs Mutated	0.63 (0.06-6.16)	0.70

HR: hazard ratio.

## 3 讨论

随着现代医学的发展，医工结合提高了医疗服务的效率和质量。在肿瘤学领域，ML可以在大规模临床研究中发现新的生物标志物和治疗靶点，推动肿瘤学研究的进展。ML技术在肺癌研究领域也被不断发掘，如通过分析呼吸相关特征或代谢指标探索潜在的肺癌生物标志物^[13]^，或从医学影像中提取深层次特征用于预测肺癌的发生及其相关基因突变^[14]^，亦或是借助ML算法对细胞图像进行分析来预测肺癌的基因突变情况^[15]^。这些方法丰富了ML在肺癌诊疗领域的应用。本研究则基于血液流网络，首先采用无监督学习算法对血液特征进行聚类，根据不同的聚类结果使用ML算法进行特征选择。然后利用互信息值量化每个特征与目标变量之间的相互依赖性，确保所选特征能够有效预测目标变量，并基于互信息值对特征进行排序，筛选出性能最佳的50个特征。接着，将筛选后的特征使用XGBoost算法进行训练。这种高效的梯度提升框架，能够捕捉到大规模数据中的复杂模式并提供准确的预测结果，此外加入正则化项以防模型出现过拟合。在模型训练完成后，通过对所有提取的特征及其相应的突变分类进行Shapley近似值分析，确定贡献最高的前10个特征及其各自的预测概率并进行预后分析。最后，通过交叉验证和ROC曲线验证了模型的预测性能。此外，利用Shapley值对模型的预测结果进行解释，确保模型的可解释性和透明度。这一系列的步骤不仅提高了模型的预测准确性，也增强了对模型决策过程的理解。

Yang等^[16]^的研究最先对外周血指标预测肺癌患者的EGFR突变状态进行了分析。该研究使用多种ML算法，发现随机森林模型在预测EGFR突变方面表现最佳。本研究ML模型在此基础上进行优化，首先，采用无监督学习聚类算法来识别血液特征中的内在模式，有助于在特征选择之前更好地理解数据结构。其次，引入更先进的特征选择和特征提取方法，如互信息值和主成分分析，以提高模型的预测精度和稳定性。此外，本研究采用XGBoost算法通过交叉验证和优化超参数，提升了模型的鲁棒性和泛化能力。同时，使用蒙特卡洛近似来估计Shapley值，这为模型的可解释性提供了更深入的理解。最后，通过交叉验证和ROC曲线的模型性能验证，确保了模型的稳定性和诊断价值。本研究结合多模态数据，包括临床数据和外周血数据等，进一步增强了模型的预测能力。最后，通过生存分析评估了生物标志物对患者预后的影响，为临床医生提供了更全面的决策支持。

既往研究^[17]^表明EGFR突变在腺癌中更常见，尤其是在亚洲从未吸烟的肺腺癌患者中，EGFR突变比例可高达60%-70%。根据PIONEER研究^[18]^，亚洲地区初诊为IIIB/IV期肺腺癌患者中，EGFR突变比例高达51%，且组织学类型与EGFR突变频率显著相关（P=0.016），这与本研究的结果相契合。此外，携带EGFR L858R突变和间质上皮细胞转化因子（mesenchymal epithelial transition factor, MET）扩增的肺腺癌患者血液嗜酸性粒细胞增多，且与疾病的进展平行，即血液嗜酸性粒细胞增多对驱动因子阳性NSCLC的预后有负面影响^[19]^。本研究同样表明嗜酸性粒细胞可作为EGFR突变的预测指标，但其在预后预测方面没有统计学差异。一项有关阿米伐他单抗（Amivantamab）的研究^[20]^证明单核细胞和巨噬细胞通过胞吞作用，能够有效下调EGFR/cMet通路。当EGFR突变型细胞在EGFR-TKIs处理后与抗CD24抗体共同培养时，单核细胞衍生的巨噬细胞促进了抗体依赖性细胞吞噬作用^[21]^，CD24表达在EGFR突变细胞中显著上调，但在EGFR野生型细胞中没有这种现象。这些研究为嗜酸性粒细胞及单核细胞反映肺癌EGFR突变的状态提供了理论依据。EGFR突变会持续激活MEK/ERK途径，而高水平的细胞外磷酸盐能够刺激细胞外信号调节这一途径^[22]^。磷酸盐水平的变化可作为反映EGFR突变状态的生物标志物。近年来的研究^[23]^揭示离子通道在癌症发展中的关键作用，特别是电压门控钠通道和细胞内氯通道。离子通道不仅调控细胞膜电位和离子平衡，还在癌症的增殖、迁移和侵袭过程中起重要作用，特别是在更具侵袭性和转移性的癌症中。钠钾ATP酶是癌症信号传导的关键因素。研究^[24]^显示，阻断钠离子载体或钠钾ATP酶导致的钠离子内流增加，可使EGF通过细胞内钠介导的组蛋白去乙酰化酶6失活和微管蛋白乙酰化调节EGFR的转运，因而钠钾水平可能间接反映EGFR突变状态。癌细胞可激活凝血系统，其与肿瘤进展密切相关。既往研究^[25]^已证实血小板计数、凝血功能和单核细胞在预测EGFR-TKIs治疗肺癌预后中的关键作用。APTT、内生肌酐清除率、单核细胞和稳态血浆谷浓度与肿瘤耐药性和侵袭性通路显著相关。其中，APTT升高与EGFR-TKIs耐药和肿瘤存活率的基因突变（如mTOR通路）相关^[26]^。

总而言之，本研究选择的特征中，病理类型、嗜酸性粒细胞、单核细胞和总胆固醇已被临床证实与基因突变相关^[19⇓-21,27]^，临床检出率为50%。这一实验结果有力地证明了多模态可解释性诊疗研究在临床实践中的重要指导意义。尽管本研究取得了一些重要发现，但仍存在一定的局限性。首先，本研究的数据集来自单一中心，样本规模相对有限，可能影响结果的普适性。未来研究可考虑扩充样本量，整合多中心数据，以验证和扩展本研究的发现。纳入研究的EGFR突变类型为最经典的两种突变，其他类型突变的预测指标与预后可能会有所偏差。其次，在实际临床实践中，患者接受的后续治疗方案多种多样，这些方案的选择和效果对患者的长期预后有显著影响。但由于回顾性研究的限制，无法获得所有患者完整的治疗历程，包括二线或三线治疗的具体方案和疗效。这限制了对患者整体治疗模式和预后影响的深入分析。在ML过程中，尽管XGBoost算法优势显著，但在特征节点分裂时需遍历整个数据集，且预排序空间复杂度高，既要存储特征值，又要存储样本梯度统计值索引。因而将数据降维处理，通过确定各指标的最佳截断值，使用ROC曲线对模型预测能力进行评估，并对前10个指标进行生存曲线分析。最后，本研究主要基于ML模型的分析结果，虽然这些模型在预测性能上表现良好，但仍需扩大样本量，并纳入多中心数据及进行前瞻性研究、外部队列验证等手段以验证和扩展本研究ML模型识别出的生物标志物的临床应用价值，推动其在实际诊疗中的应用。

综上，本研究使用ML开发了一种基于外周血指标预测EGFR突变的新型预测模型。该模型对传统算法进行优化，使用了较为先进的算法，具有高预测精度、良好的稳定性和较强的泛化能力等优点，为欠发达地区及无法进行基因检测的患者提供较为科学的依据，帮助临床医生在资源有限的情况下进行个体化治疗方案的制定。未来的研究应进一步验证和优化该模型，并探索其在多中心和更大样本量中的应用，以提高其临床实用性和普适性。
